# Pharmacokinetic-pharmacodynamic modeling of tylosin against *Streptococcus suis* in pigs

**DOI:** 10.1186/s12917-018-1645-3

**Published:** 2018-10-24

**Authors:** Lingli Huang, Haiyang Zhang, Mei Li, Ijaz Ahmad, Yulian Wang, Zonghui Yuan

**Affiliations:** 1 MOA Laboratory for Risk Assessment of Quality and Safety of Livestock and Poultry Products, Wuhan, Hubei China; 2 National Reference Laboratory of Veterinary Drug Residues (HZAU) and MOA Key Laboratory for the Detection of Veterinary Drug Residues in Foods, Wuhan, Hubei China; 3 Huazhong Agricultural University, Wuhan, Hubei China; 4 Department of Animal Health, The University of Agriculture Peshawar, Peshawar, 25130 Pakistan

**Keywords:** Tylosin, *Streptococcus suis*, Pig, Dosage regimen, PK/PD modeling

## Abstract

**Background:**

The aim of this study was to optimize the dosage regimen of tylosin against *S.suis* in Pigs using pharmacokinetic-pharmacodynamic (PK-PD) modeling. The antibacterial activity of tylosin against *S.suis* CVCC606 was investigated in Mueller Hinton (MH) broth and serum. The objectives of this investigation were to study the PD data of tylosin against *S.suis* CVCC606 and the PK data of tylosin in healthy and diseased model of pigs and formulate a rational dosage regimen for the treatment of pig streptococcosis.

**Results:**

The minimum inhibitory concentrations (MIC) were 0.25 μg/mL, and the minimal bactericidal concentrations (MBC) were 1 μg/mL in MH broth and serum. The killing curve showed time-dependent activity and weak concentration-dependent antibacterial activity. A pig pneumoniae model of *S. suis* infection was built by inoculating subcutaneously with *S. suis* CVCC606. Tylosin was (10 mg/kg b.w) administered intramuscularly (IM) to the healthy and *S.suis* infected pigs, The pharmacokinetic properties, including area under the curve(AUC), peak concentration (C_max_) and time to reach C_max_ (T_max_), were determined in plasma using UV-HPLC method. The AUC, C_max_ and T_max_ in plasma of healthy and infected pigs were 10.80 ± 2.20 and 10.30 ± 3.46 μg.h/mL, 2.06 ± 0.43 and 2.37 ± 0.38 μg/mL, 1.95 ± 0.22 and 1.58 ± 0.49 h, respectively.

**Conclusions:**

The in vivo PK and in vitro PD data were integrated to determine the surrogate marker of antibacterial activity, *C*_max_/MIC, AUC/MIC and T_>MIC_were 8.90, 43.21, 8.86 for healthy pigs, and 9.76, 41.18, 7.56 for infected pigs, respectively. Ex vivo AUC/MIC data were integrated with ex vivo bacterial count to calculate the values for bacteriostatic and bactericidal action, which were 10.67 h and 49.66 h for healthy pigs, 11.73 h and 43.03 h for pigs infected with *S.suis*. A dosage regimen of 5.32–19.50 mg/kg b.w. every 24 h should be sufficient for tylosin against *S.suis*.

## Background

*Streptococcus suis* is a Gram-positive facultative anaerobe and increasingly emerging zoonotic infection with a global distribution [1] . The most common clinical syndrome casued by *Streptococcus suis* are porcine meningitis, encephalitis, pneumonia, endocarditis, polyserositis arthritis and septicemia [2, 3]. Among the 35 serotypes of *S. suis*, serotype 2 is a predominant isolate from diseased animals and humans [4–6]. This microorganism is responsible for causing diseases in various species including human, mammals and birds. Contaminated raw and undercooked pork is the major source of transmission of this organism [7, 8]. *S. suis* has negative impact on pig industry and causes severe economic losses. *S. suis* is usually treated by Tetracyclines and Macrolides in veterinary medicine, but an increasing emergence of resistance against Tetracyclines has been widely reported in recent years [9], so Macrolides are widely used for the treatment of infection caused by *S. suis*.

Tylosin belongs to the group of 16-member-ring macrolides, which was first derived from *Streptomyces fradiae* cultures in 1960 [10]. Due to its bacteriostatic action against Gram-positive bacteria, anaerobic bacteria and *Mycoplasmas* [11], tylosin has been widely used to treat pneumonia, arthritis, respiratory tract infections, porcine streptococcosis and other infections in veterinary medicine [12, 13]. Tylosin is widely used as antimicrobial agent in China and administered through pareteral or oral route. The Pharmacokinetics study of tylosin has been described in a variety of animals including hens [14], broiler chickens [15], goats [16], cows [17] and dog [18]. However, there are limited data available on the Pharmacokinetics/pharmacodynamics model of tylosin in pigs [19].

PK-PD model is widely used in the determination of a dosage regimen for an antimicrobial, which can reflect the relationship of drug, bacteria and animals. The impetus to optimize dosage schedules of antimicrobial drugs has been driven not only by increased knowledge of bacterial killing mechanisms, which may be concentration-dependent, time-dependent or co-dependent, but also by the results of laboratory animal studies, target species investigations and clinical trial outcomes [20].

In this investigation, the PK data of tylosin were examined in healthy and diseased model of pigs. The purposes of the study were (1) to establish the experimental model of porcine streptococcosis, and determine PK data of tylosin in healthy and *S. suis* infected pigs after IM administration at a dose of 10 mg/kg. (2) To investigate the in vitro PD data of tylosin against *S. suis* in MH broth and serum, and investigate the ex vivo activity of tylosin in serum. (3) To combine MSW theory with traditional PK-PD model using the parameters (T_>MPC_, T_MSW_ and so on) to predict the emergence of the bacterial resistance. It is proposed that these parameters were used to formulate a rational dosage regimen for the treatment of pig streptococcosis, which will provide maximal efficacy and minimal opportunity for the emergence of bacterial resistance.

## Results

### Pharmacodynamics

#### MICs, MBCs and MPC of tylosin against *S. suis* CVCC606

The MIC values of tylosin against the strain of *S. suis* CVCC606 were 0.25 μg/mL, and MBC values are 1 μg/mL in MHB and serum obtained from pigs. MPC value of tylosin against *S. suis* CVCC606 was 1 μg/mL, MSW was 0.25–1 μg/mL, explaining the resistant mutant selection window is narrow. We found that in the presence of serum, the antimicrobial effect of tylosin in serum was the same as in broth. So it had a great clinical significance to study the antimicrobial effect of tylosin in vivo.

#### Time-kill curve

**Fig. 1:**
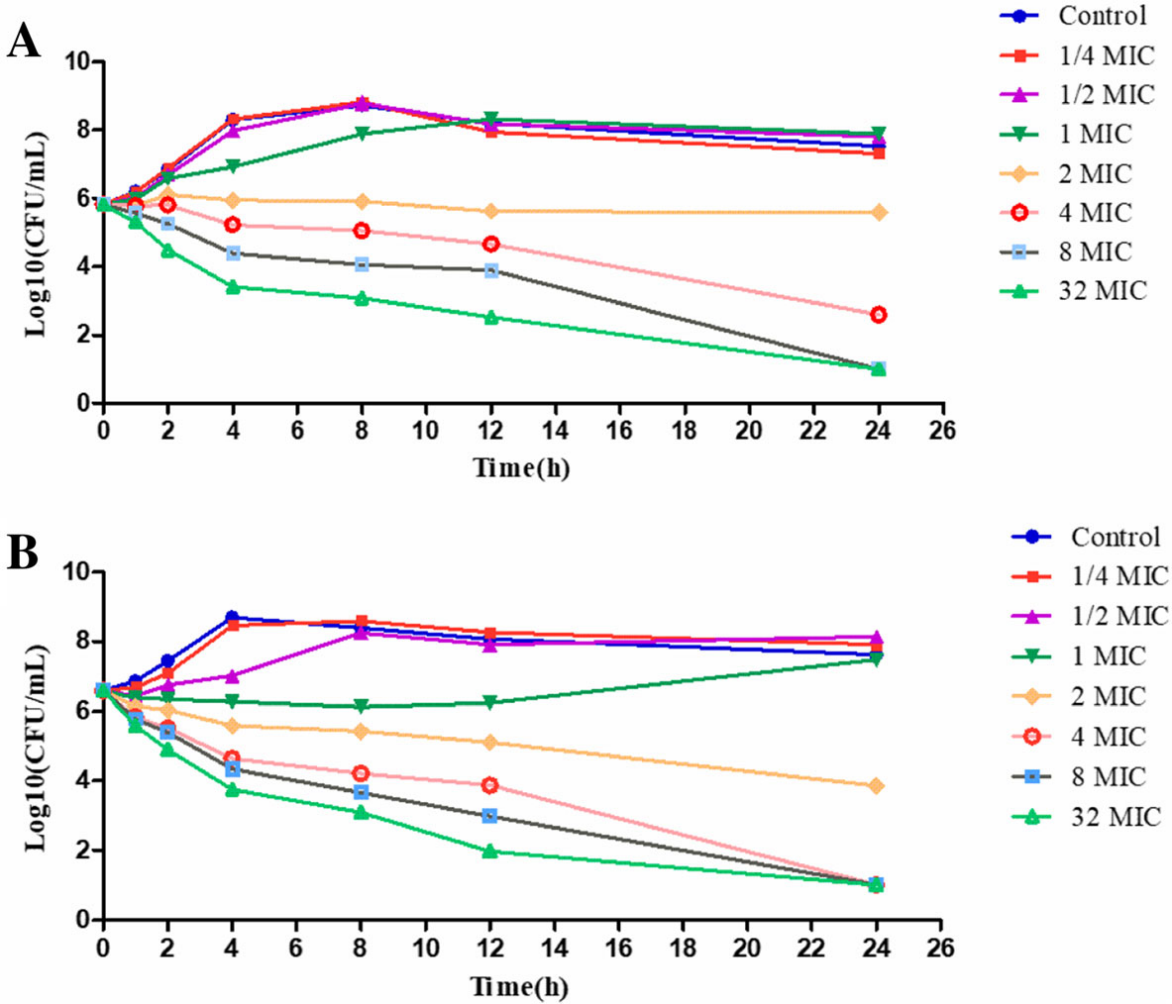
Killing curve of tylosin against *S. suis* in broth (**a**) and serum (**b**) measured at pre-determined time. The x-axis was the 0–24 h incubation time point; y-axis was the count numbers exposed to a series of concentrations of tylosin

The time-dependent feature of tylosin against *S. suis* was demonstrated according to the killing profiles (Fig. 1), the increasing rate of killing was observed by increasing the time that *S. suis* was exposed to tylosin, meanwhile the killing profiles also showed a weak concentration-dependent feature, and the increasing rate of killing was observed by the increasing concentration of tylosin.

#### PAE of tylosin against *S. suis* CVCC606

The PAEs for tylosin against *S. suis* were displayed in Table 1, which showed that the durations of PAE were directly related to the exposure time, it lasted longer when the bacteria were exposed to tylosin for 2 hours than those for 1 hour.

**Table 1:** PAEs of tylosin against *S. suis* CVCC606

Concentration	Expose 1 h	Expose 2 h
MIC	0.21	1.80
2MIC	1.43	3.43
4MIC	2.15	4.21

#### Ex vivo antibacterial activity of tylosin against *S. suis* CVCC606

**Fig. 2:**
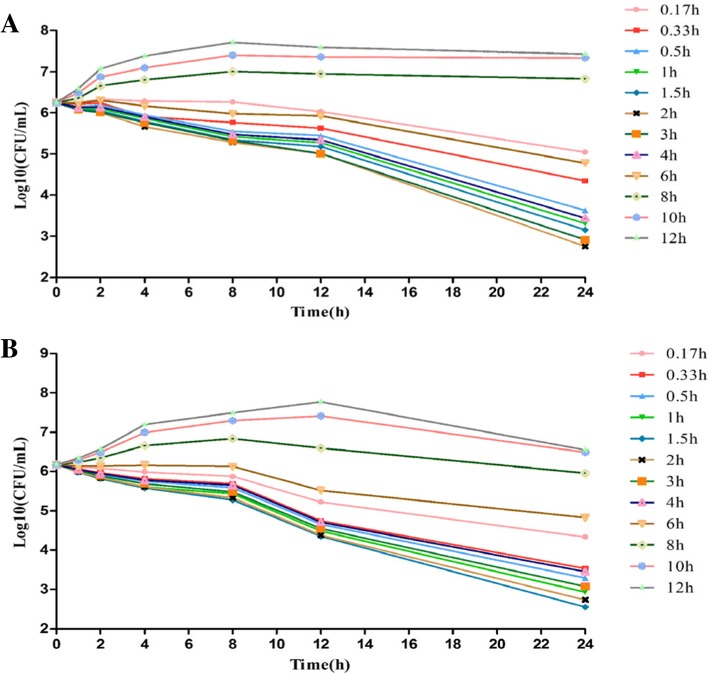
The ex vivo antibacterial curve of tylosin against CVCC606 in serum from healthy pigs (**a**) and diseased pigs (**b**). The ex vivo antibacterial activity in serum was determined in samples harvested at pre-determined times (0.17, 0.33, 0.5, 1, 2, 3, 4, 6, 8, 10, 12 h after tylosin IM dosing)

For serum samples collected from all healthy pigs at 0.5, 1, 1.5, 2, 3 and 4 h, tylosin resulted in a > 3log_10_ reduction in viable bacterial count after 24 h of exposure (Fig. 2). For samples collected at 0.17, 0.33 and 6 h, tylosin resulted in a < 2log_10_ reduction in viable bacterial count after 24 h of exposure. No bacteriostatic or bactericidal effects were observed from serum samples collected at 8, 10 and 12 h. For serum samples collected from all pigs with *S.suis* at 0.33 h, tylosin exerted a very strong bactericidal effect, and the other samples were similar to the samples collected from healthy pigs.

### Pharmacokinetics

#### Experimental model of porcine streptococcosis

When the experimental model of the porcine streptococcosis was successfully established, the pigs exhibited obvious clinical symptoms, such as roughened body coats, loss of appetite, elevated body temperature (40.0 to 42.0 °C), and were reluctant to rise and lame in one or more legs, a few of them exhibited severe central nervous system signs such as head tilt, nystagmus, tremors, prostration and opisthotonus.

#### Pharmacokinetics of tylosin in pigs

**Fig. 3:**
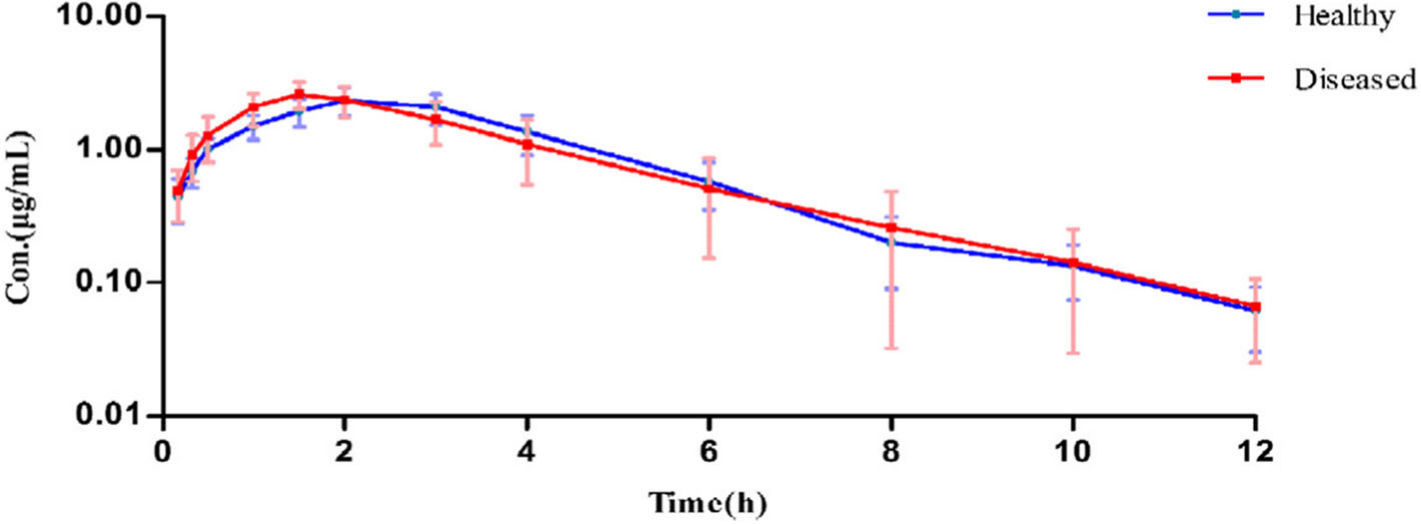
Semi-logarithmic plot of serum concentrations of tylosin after IM administration at a dose rate of 10 mg/kg b.w. (*n* = 8)

Serum concentrations of tylosin after IM dosing in healthy pigs and diseased pigs were illustrated in Fig. 3. Tylosin concentration-time profiles in serum were described by mono-compartmental with a first order absorption phase in all pigs.

The PK parameters of tylosin were illustrated in Table 2. Absorption and elimination of tylosin after IM administration at a dose rate of 10 mg/kg were rapid in both healthy pigs and diseased pigs, T_1/2_k_a_ were 1.347 h and 1.060 h, T_1/2_k_e_ were 1.354 h and 1.152 h, respectively. Means *C*_max_ of 2.056 μg/mL (healthy) and 2.372 (*S. suis* infectious) were reached at 1.948 h and 1.548 h. The area under concentration-time curve (AUC) in both healthy pigs (10.804 h·μg/mL) and diseased pigs (10.297 h·μg/mL) were similar. The differences in K_a_, K_e_, T_1/2_k_a_, T_max_ and *C*_max_ were significant (*P* < 0.05). The fast elimination of tylosin from serum was indicated by the values of MRT.

**Table 2:** Pharmacokinetic parameters of tylosin (*n* = 8) after 10 mg/kg IM administration in pigs

Parameter	Unit	Healthy (mean ± SD)	Porcine streptococcosis (mean ± SD)
k_a_	1/h	0.521 ± 0.061	0.759 ± 0.372*
k_e_	1/h	0.518 ± 0.060	0.634 ± 0.152*
AUC	h·μg/mL	10.804 ± 2.204	10.297 ± 3.458
T_1/2_k_a_	h	1.347 ± 0.155	1.060 ± 0.383*
T_1/2_k_e_	h	1.354 ± 0.150	1.152 ± 0.293
T_max_	h	1.948 ± 0.219	1.578 ± 0.487*
C_max_	μg/mL	2.056 ± 0.426	2.372 ± 0.376
AUMC	h·h·μg /mL	36.169 ± 6.570	35.531 ± 17.647
MRT	h	3.588 ± 0.469	3.353 ± 0.694
CL/F	mL/min	15.944 ± 2.946	18.1326 ± 7.265

Pharmacokinetics parameters and variables were calculated using a one-compartment model with first order input and output: k_a_ is absorption rate constant; k_e_ is elimination rate constant; T_1/2_k_a_ is absorption half-life; T_1/2_k_e_ is elimination half-life; C_max_ is maximum concentration in serum; T_max_ is the time to achieve the maximum serum concentration; AUC is area under serum concentration-time curve; AUMC is area under the first moment curve; MRT is mean residence time; CL/F is the body clearance corrected for bioavailability

*means significance difference (*P* < 0.05)

### PK-PD modeling

#### In vivo PK-PD parameters

Integration of in vivo PK and in vitro PD data of tylosin were represented in Table 3 as the indices AUC_24h_/MIC, AUC_24h_/MBC, *C*_max_/MIC, *C*_max_/MBC, T_>MIC_, T_>MBC_. The mean AUC/MIC ratios in serum collected from the healthy pigs and diseased pigs were 43.216 and 41.188 h, respectively. *C*_max_/MIC ratios were 8.900 and 9.768, T_>MIC_ were 8.863 and 7.568 h, respectively.

**Table 3:** In vivo PK-PD parameter of tylosin afer IM administration at a dose rate of 10 mg/kg (*n* = 8)

PK-PD parameter	Unit	Healthy Pigs Mean ± SD	Infected Pigs Mean ± SD
AUC	h·μg/mL	10.804 ± 2.204	10.297 ± 3.458
C_max_	μg/mL	2.225 ± 0.485	2.442 ± 0.389
MIC	μg/mL	0.250	0.250
MBC	μg/mL	1.000	1.000
AUC_24 h_/MIC	h	43.216 ± 8.816	41.188 ± 13.832
AUC_24 h_/MBC	h	10.804 ± 2.204	10.297 ± 3.458
C_max_/MIC	–	8.900 ± 1.940	9.768 ± 1.556
C_max_/MBC	–	2.225 ± 0.485	2.442 ± 0.389
T_>MIC_	h	8.863 ± 0.914	7.568 ± 2.220
T_>MBC_	h	4.712 ± 0.756	4.327 ± 1.433

#### Ex vivo PK-PD parameters

The ex vivo AUC_24h_/MIC ratios of tylosin were presented in Table 4 after IM administration at a dose rate of 10 mg/kg. The ex vivo AUC_24h_/MIC were calculated by dividing the AUC values with in vitro MIC values. E was calculated by counting the change in the bacterial count (log10 cfu/mL) in the serum sample harvested from different time points.

**Table 4:** Ex vivo AUC_24h_/MIC value (mean ± SD, *n* = 8) of tylosin after IM administration at a dose rate of 10 mg/kg

Time(h)	Healthy Pigs		Infected Pigs	
	AUC_24 h_/MIC(h) (Mean ± SD)	E	AUC_24 h_/MIC(h) (Mean ± SD)	E
0	0	3.23	0	3.345
0.17	10.587 ± 3.835	−1.199	11.832 ± 4.979	−1.833
0.33	16.617 ± 4.260	−1.895	22.185 ± 8.413	−2.633
0.5	24.177 ± 5.010	−2.614	30.768 ± 11.325	−2.879
1	35.904 ± 7.518	−2.932	49.743 ± 13.588	−3.234
1.5	46.572 ± 11.173	−3.085	62.532 ± 14.423	−3.620
2	56.271 ± 13.789	−3.488	56.343 ± 14.827	−3.431
3	49.797 ± 13.062	−3.329	40.374 ± 14.276	−3.091
4	32.676 ± 10.897	−2.800	26.565 ± 13.558	−2.716
6	13.854 ± 5.403	−1.462	12.162 ± 8.473	−1.338
8	4.794 ± 2.640	0.587	6.189 ± 5.428	−0.212
10	2.712 ± 1.409	1.093	3.393 ± 2.693	0.319
12	1.452 ± 0.750	1.184	1.578 ± 0.979	0.382

#### Sigmoid *E*_max_ model

**Fig. 4:**
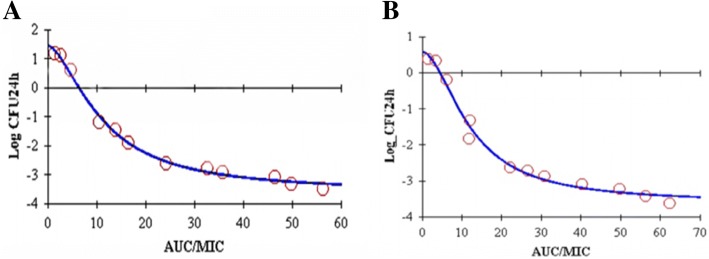
Sigmoid Emax relationship for bacterial count vs. ex vivo AUC/MIC in serum from healthy pigs(**a**) and diseased pigs(**b**)

Data derived from PK-PD modeling of the ex vivo growth inhibition curves were presented in Table 5 and Fig. 4. For serums of healthy pigs and diseased pigs, the values of *E*_max_ of healthy and diseased pigs were 3.23 and 3.345, respectively. *E*_max_ values were similar and indicated a high level of attainable bacterial killing in serum.

**Table 5:** The result of the sigmoid *E*_max_ model

Parameter	Healthy	porcine streptococcosis
*E*_max_	3.230	3.345
EC_50_	11.171	12.233
E_0_	−3.488	−3.620
N	1.707	1.849
*E*_max_-E_0_	6.718	6.965
AUC_24 h_/MIC for bacteriostatic action	10.679	11.736
AUC_24 h_/MIC for bactericidal action	49.665	43.032

*E*_max_ is maximam difference in log_10_ of bacterial number of sample incubated with drug, EC_50_ is the PK-PD parameter of drug that produce 50% of the maximal antibacterial effect, E_0_ is the difference after 24 h incubation in log_10_ of number of bacteria in control samples, N is the HILL coefficient which discribes the steepness of the parameter-effect curve

#### Dosage regimen

Parameter values and corresponding dosage of tylosin achieving different antimicrobial activity were exhibited in Table 6. The value of ex vivo AUC/MIC obtained for bacteriostatic action in serum from diseased pigs was 11.736. Hence, for the MIC of 0.25 μg/mL, the lowest dose providing bacteriostatic activity is 5.320 mg/kg, the calculated dose for antibacterial activity of killing 99.9% of *S. suis* strains is 19.507 mg/kg, which the corresponding value of AUC/MIC was 43.032, assuming a dosage interval of 24 h. According to the calculation, For the clinical application of tylosin against *S. suis*, the dosage regimen of 5.320–19.507 mg/kg every 24 h for IM administration was recommended.

**Table 6:** AUC/MIC parameter values and dosage achieving different antibacterial effect based on infected pigs

Antibacterial effect	AUC/MIC Values	Dosage (mg/kg)
Bacteriostatic	11.736	5.320
Bactericidal	43.032	19.507

#### Resistance risk assessment of tylosin

The results were presented in Table 7, and showed that drug-resistant strains don’t appear selectively when the concentration of tylosin was lower than 0.25 μg/mL. However, when the drug concentration was in the range of 0.25-1 μg/mL, drug-resistance strains had selective growth and the growth of sensitive strains was inhibited. The drug-resistant strains do not appear when the concentrations of tylosin are higher than 1 μg/mL.

**Table 7:** The growth of resistant strains after exposed to different concentrations of tylosin

Concentrations (μg/mL)	Time(h)					
	0	2	4	8	12	24
0	+	+	+	++	++	+
0.125	+	+	+	+	++	++
0.25	+	+	+	+	++	++
0.5	+	+	+	++	++	+
1	+	+	+	+	+	+
2	+	+	+	+	N	N
4	+	+	N	N	N	N
8	+	+	N	N	N	N

N, colony number below 10 cfu/mL; +, colony number between 10 and 1000 cfu/mL; ++, colony number beyond 1000 cfu/mL

## Discussion

Dosage regimens of a drug were established according to the PD data and PK data from healthy and infected animals. However, the physiological status of the diseased animals should be considered when the dosage regimens were established. When animals were infected, body temperature, organization blood flow, capillary permeability, metabolic ability, plasma protein binding ratio etc. would be changed. The changes of the physiological status could influence the ADME, In diseased animals the Pharmacokinetics characteristics of the drug would be different from healthy animals [21]. So we studied the Pharmacokinetics of tylosin in diseased pigs, and compared the character with healthy pigs. In this investigation, the experimental model of porcine streptococcosis was established through the subcutaneous inoculation [22]. Dosage regimen was established according to the PK data and PD data of tylosin, which were derived from diseased animals.

The PK of tylosin had already been investigated in goat, sheep, pigs, chickens [23] and camel [24] following intravenous(IV) and/or IM administration. In the present study, the result showed that tylosin fitted the one-compartmental open model in healthy pigs and diseased pigs in accordance with previous reports in different animals [25]. The absorption half-life of tylosin in diseased pigs was slightly shorter than that reported in healthy pigs (T_1/2ka_ = 1.36 h) [26]. In our study the terminal half-life was shorter in both diseased (T_1/2ke_ = 1.152 h) and healthy pigs (T_1/2ke_ = 1.354 h) than previous investigations in pigs (T_1/2ke_ = 3.01 h), cattle and buffaloes (2.24 and 2.4 h, respectively) [27]. A higher *C*_max_ values were obtained in diseased pigs (2.372 μg/mL) than healthy pigs (2.056 μg/mL), but both were lower than that found in previous data (*C*_max_ = 2.71 μg/mL). This study showed that the time to reach the maximum concentration of tylosin in blood is short in diseased animals as compared to healthy pigs. In this study the PK parameters T_1/2ka_ and T_max_ were significantly lower in diseased pigs than healthy pigs. The results indicated that tylosin had a quicker absorption and elimination in infected pigs with *S. suis*, and tylosin reached the peak concentration earlier. Therefore, it had a great clinical significance that to formulate a dose schedule of tylosin against porcine streptococcosis.

The differences of the Pharmacokinetics of tylosin in healthy and diseased pigs may be due to the change of the physiological and biochemical indices, such as the change of body temperature, the decrease of the protein in plasma, the decline of plasma glue through pressure, anemia, liver dysfunction of acetylation and so on. The changes can influence the absorption, distribution and elimination of the drug in animal.

The determination of MIC is the lowest concentration that the drug can inhibit the growth of microorganism in an artificial medium, such as in agar and in broth. The composition of the artificial medium is different from serum in several respects, such as the electrolyte concentrations (calcium and magnesium), pH and protein concentration. Consequently, The MIC value determined in broth or agar can’t take the place of those determined in serum. For example, Pridmore determined the range of MICs for Tiamulin against 4 strains of Actinobacillus pleuropneumoniae in culture and serum were 12–24 μg/mL, 14–24 μg/mL, 12–32 μg/mL and 12–24 μg/mL, respectively [28], showed that tiamulin had different antibacterial effect in culture and in serum. However, in this investigation, the MIC of tylosin against *S. suis* strains in MH broth and serum were the same 0.25 μg/mL.

The ex vivo antimicrobial data were generated into Sigmoid *E*_max_ equation, indicated that tylosin could achieve the bacteriostatic and bactericidal action, but couldn’t achieve the elimination action. The reason of this phenomenon was that tylosin is a bacteriostatic drug, which inhibits growth of the organism and requires the aid of defense system to clear the infecting microorganisms of tissues. For a bacteriostatic drug, when drug levels are lower than MIC, the decline of bacterial count is mainly the result of the host defense. But the rate and degree of bacteriostatic drugs against microorganisms is weaker than bactericidal drugs. Aliabadi established PK-PD model of danofloxacin against *Mannheimis haemolytica*, and the *E*_max_ of danofloxacin was 4.967, which could achieve bactericidal action and elimination action [29]. For bactericidal drugs, they showed an obvious effect of sterilization when the concentration is above MBC after animals were administrated, and they showed a bacteriostatic effect when the concentration is below MBC.

Macrolides is a kind of time-dependent drug, but every drug has its own characteristic. The conventional macrolides (e.g. erythromycin) are classical time-dependent drugs without PAE [30]. However, a few of them (e.g. azithromycin) express time-dependent and weak concentration-dependent with prolonged PAE. So one parameter (T_>MIC_) can’t describe the antibacterial activity of macrolides.

Tylosin killed *S. suis* by a time-dependent and weak concentration-dependent characteristic with prolonged PAE, the antimicrobial effect was strengthened with the extension of time or the increase of concentration. The PAE of tylosin against *S. suis* was prolonged, the PAE was 3.43 h when *S. suis* was exposed in 2MIC for 2 h, and 4.21 h when was expose in 4MIC for 2 h. So the the best PK-PD index responsible for the efficacy of of tylosin against *S. suis* was AUC/MIC according to the bacterial killing curve and PAE.

It is necessary to consider whether the differences in ex vivo conditions used in this investigation and those occurring clinically in diseased animals may be associated with differences in tylosin efficacy. First, the role of host defense mechanisms must be regard as a primary consideration in vivo. For the healthy animal the immune system is competent, so the body’s defense mechanisms playing an auxiliary function and will exert synergistic effect with administered drug. However, host defense mechanisms are not taken into consideration under in vitro conditions. A second difference is that a single strain was used in ex vivo investigation, but in vivo study, several strains are in the body at the same time. The predictions of dosage treating with this organism are likely to act on other strains of the same organism, but this can’t be assumed for acting on other species of bacteria. Thirdly, under ex vivo condition, organisms are exposed to a fixed drug concentration for a fixed time to carry out the assessment of bacterial count made at 1, 2, 4, 8, 12 and 24 h. However, under in vivo conditions, the concentrations of the drug first raise to a peak and then decrease, the concentration will keep changing unless the drug is infused IV at a rate such that the serum concentration will maintain constant (the infusion using a precise rate can ensure that administration and elimination rates are exactly balanced).

The PK-PD indices derived from serum were integrated using the present data, the parameter of AUC/MIC in serum relates to the biophase, which is with regard to bacteria in plasma and tissues. The data derived from serum are more relevant to the host conditions than in vitro investigation of using artificial media such as MHB. The growth curve of the bacteria was also determined in serum, which is more appropriate than in vitro PD parameters (such as MIC, MBC and the killing curve), to describe the antibacterial activity of tylosin. MIC and MBC are indirect or surrogate maker, but they are essential because direct indices of antimicrobial activity are not available.

The rationale of using the AUC/MIC to calculate the rational dosage is that Pharmacokinetics of the drug in vivo expressing a linear kinetic character, the rate of the drug’s transformation and elimination are dose-independent and concentration-independent. There is positive correlation between AUC and/or the concentration of the drug in plasma or serum. According to our PK and PD parameters in this study, the optimal single dose required to reach bacteriostatic, bactericidal activity were 5.320 and 19.507 mg/kg, respectively. What’s more, tylosin was a growing bacteriostatic drug which exerted its function by inhibiting protein synthesis of the organism, and could reach bacteriostatic and bactericidal action. The dose regimen was depended on bacterial population and PK data obtained from diseased animal, this might be more appropriate to take into account the conditions of diseased pigs in clinical application.

According to the theory of MSW, when the concentration of drug is below MIC, the growth of bacteria is not inhibited, and drug-resistant mutant strains are not dominant in the growth of the whole bacteria. When the concentration of drug is in the range of MIC-MPC, the sensitive bacteria are inhibited and the drug-resistant strains can grow selectively, thus bacterial resistance develops. Assessment of bacteria resistance could be performed by PK-PD model theory and MSW theory. Based on our results, the times that tylosin concentrations in pigs maintained above 1 μg/mL (T_>MPC_) were 4.712 h and 4.327 h for healthy pigs and *S. suis* infected pigs, and *C*_max_ were 2.056 μg/mL and 2.372 μg/mL, respectively. And the times that tylosin concentrations in pigs from 1 μg/mL to 0.25 μg/mL (T_MSW_) were 3.773 h and 2.997 h for healthy pigs and *S. suis* infected pigs, respectively. The PAE that *S. suis* were exposed in 1 μg/mL tylosin solution for 2 h was 4.21 h, So the bacteria were inhibited when the tylosin concentration was in MSW, and no selective growth of bacteria were produced, therefore no resistance emerge after IM administration at a dose rate of 10 mg/mL.

## Conclusions

According to our investigation the tylosin had the same antibacterial activity in both serum and MH broth, and showed an obvious time-dependent and weak concentration-dependent antimicrobial activity, therefore the best PK/PD surrogate marker was AUC/MIC. Porcine streptococcosis for the dosage regimen, the calculation of dosage for prevention and treatment based on the data of diseased pigs, and it might be more practical to apply clinically for tylosin against *S. suis*. The dosage regimen of 5.320–19.507 mg/kg b.w. for every 24 h should be adequate for the treatment of tylosin against *S. suis* in clinical practice.

## Methods

### Antimicrobial

Tylosin standard was purchased from Dr. Ehrenstorfer Germany for in vitro experiments (Det. Purity 98%; Lot Number 17895600). Tylosin injectable solution (50 mg/mL) was compounded before experiment; each milliliter contains 50 mg of tylosin activity (as tylosin base) in 50% propylene glycol with 4% benzyl alcohol and water for injection.

### Bacteria

*S. suis* CVCC606 (pig isolate, serotype 2) was purchased from Chinese veterinary culture collection. The strain was grown freshly from beads, previously store at − 70 °C, on tryptone soya blood agar.

### Animals

The study was carried out on 16 pigs (Duroc × Large White × Landrace pigs) of either sex which were 6 weeks old having average weight of 16 ± 2 kg. The animals were acclimatized for a period of 1 week before experiment. Animals were housed in two separated concrete floor rooms, fed twice daily and watered ad libitum. The Euthanasia procedure is carried by pentobarbital sodium with IV administration when study was finished. The experimental procedures involving animals in the study were approved by the Animal Ethics Committee of Huazhong Agricultural University and the Animal Care Center, Hubei Science and Technology Agency.

After acclimatization period the animals were divided randomly into Group A and B. Group A was inoculated subcutaneously with 1 mL of 1.2 × 10^9^ cfu/mL *S. suis* to establish the disease model. The animals in group B were kept as control.

After the inoculation, further experiment was started when streptococcosis symptoms like high temperature, loss of appetite, spiritual malaise, breathing rate increased, coughed, corneal flushed, joints swelling, CNS signs were observed.

### In vitro pharmacodynamic of tylosin against *Streptococcus suis* CVCC606

#### Determination of MIC, MBC in broth and serum

MIC (Minimal Inhibitory Concentration) was defined as the minimum concentration of drugs where no visible growth of bacteria was observed. The determination was performed by microbroth dilution method according to CLSI (Clinical and Laboratory Standards Institute, formerly NCCLS) document VET01 A4, 2013. A logarithmic phase culture of each bacterial strain was diluted with proper broth in order to obtain a density of 1 × 10^6^ cfu/mL.

Tylosin solutions containing a 128 μg/mL of Tylosin were added to 0.1 mL of MHB or serum (obtained from the control animals). Serial dilutions were prepared in broth or serum with concentrations ranging between 64 μg/mL and 0.0625 μg/mL, and dilutions were prepared in 96-wells microplate. Plates were inoculated with 0.1 mL of culture to give a final concentration of approximately 5 × 10^5^ cfu/mL. Plates were incubated at 37 °C for 18–24 h, then shaken to mix the contents. After the tylosin-inoculum mixture was mixed, plates were incubated at 37 °C for 18 h.

An aliquot of 100 μL from each tube was subcultured on TSA, the plates were incubated at 37 °C overnight, and the colonies were counted, the limit of the detection was 10 cfu/mL. MIC was determined as the lowest concentration at which bacteria numbers remained at the original inoculums level. MBC (Minimal Bactericidal Concentration) is the lowest concentration where bacteria numbers were reduced by 99.9% and was determined according to the CLSI document M26-AE [31].

#### Determination of MPC

The mutant prevention concentration (MPC) was determined by agar method according to the procedure of Blondeau [32]. MPC was defined as the lowest drug concentration that prevented bacterial colony formation from a culture containing> 10^10^ bacteria. The *S. suis* were concentrated to> 10^10^ cfu/mL bacteria. 0.1 mL of the bacterial suspension (final concentration of 10^10^ cfu/mL) was cultured on MH Agar plates containing concentrations of tylosin in a series of two-fold dilutions, beginning with a concentration equal of the MIC. Inoculated plates were incubated for 72 h, and colonies were counted every after 24 h. All MPC determinations were performed in duplicate.

#### Time-kill curve

The time-kill curves were established by making different concentrations of tylosin ranging from 1/4 MIC to 32 MIC before bacterial inoculation of *S. suis* (10^6^ cfu/mL). Growth of bacteria was checked with control. The tubes containing cultures of bacteria and different concentrations of drugs were incubated under aerobic conditions at 37 °C for 24 h.The Bacterial count (cfu/mL) was checked after 1, 2, 4, 8, 12 and 24 h incubation, by re-seeding aliquots on agar medium in the absence of tylosin.

#### Determination of PAE

The post-antibiotic effect (PAE) was determined after removal of drug by dilution method. The *S. suis* were incubated with 1MIC, 2MIC and 4MIC of drug. After 1 and 2 h incubation the drug was eliminated by several times centrifugation and wash with fresh medium. Growth curves were determined for 24 h. The PAE was calculated from the regrowth curves using the equation: PAE = T-C. In which T is the time required for the bacterial population in the test culture to increase 1log_10_ after dilution, and C is the corresponding time for the control culture.

### Pharmacokinetics of tylosin in pigs

#### Dose and sampling

A pharmacokinetic study was carried out in pigs. Each pigs received tylosin at a dose of 10 mg/kg of body weight by IM administration. Blood samples (2 mL) were collected at 0, 0.17, 0.33, 0.5, 1, 1.5, 2, 3, 4, 6, 8, 10 and 12 h after tylosin administration for determination of tylosin concentration and ex vivo antibacterial activity. The samples were collected without anticoagulant, and then kept at a room temperature for 2 h in dark. Blood was centrifuged at 3000 r/min for 10 min to obtain serum, and serum samples were protected from light and stored at − 20 °C prior to the analysis.

#### HPLC analysis of tylosin in serum

Tylosin concentrations in pig serum were determined by a Waters 2695 series HPLC and a Waters 2487 UV detector set at a wavelength of 286 nm. A volume of 0.5 mL of serum was added to a 10 mL tube, then added 4 mL acetonitrile to precipitate proteins. After centrifugation at 4000 r/min for 10 min, the supernatant was collected into a tube and evaporated with a nitrogen instrument. The dry extracts dissolved in 200 μL of the mobile phase were injected into the chromatographic system after filtered. Calibration curves were prepared after adding tylosin into blank samples before they were extracted by the method described above. HPLC was performed in a reverse-phase column C18 (4.6 × 200 mm, 5 μm particle size). The mobile phase was acetonitrile: 0.1 M ammonium formate at a flow rate of 1.0 mL/min. The mean recovery of tylosin from serum samples was 93 ± 4% across a series of concentrations investigated. The limits of detection (LOD) were calculated on a signal to noise ratio of 3, and the value was 30 ng/mL, and the limit of quantification (LOQ) at signal to noise ratio of 10 was 50 ng/mL. The accuracy and precision of method was investigated with standard serum samples containing series of tylosin concentrations, and the inter-assay and intra-assay coefficients of variation were less than 10%, respectively. The specificity of the method above was suitable for these target substances, and there was also no endogenous interference on chromatograms.

#### PK analysis

Pharmacokinetic parameters and the concentration-time data of tylosin in serum from individual pigs were analyzed using the Winnonlin programme (Pharsight Corporation, Mountain View, CA, version 5.2, USA). Serum data were submitted to compartmental analysis using non-linear least squares regression. Data for serum were also subjected to non-compartmental analysis using the statistical moment approach and Winnonlin programme. The linear trapezoidal rule was used to calculate the area under concentration-time curve (AUC) and area under the first moment curve (AUMC). The mean residence time (MRT) was determined as AUMC/AUC.

#### Statistical analysis

All data were presented as means±SD. For these parameters and variables, the SDs for arithmetic means had been employed to give an indication of the variation in data. For PK variables, the statistical differences between healthy pigs and *S.suis* infected pigs data were assessed using the significant difference method with SPSS software package. Chi-squared tests were applied to determine whether there were statistical differences in the antimicrobial resistance. A *P*-value< 0.05 was considered to indicate statistical significance in the results.

### Ex vivo antibacterial activity of tylosin

*S. suis* CVCC606 was grown freshly on TSA, three to five colonies were selected to inoculate in 9 mL MHB and then the tubes were placed at 37 °C in incubator overnight. Serum samples were collected at 0, 0.17, 0.33, 0.5, 1, 1.5, 2, 3, 4, 6, 8, 10 and 12 h post administration of drug from healthy and diseased pigs. A 5 μL of bacterial culture in stationary phase was added to 0.5 mL serum, giving a final inoculum of 1 × 10^6^ cfu/mL. The tubes containing bacteria and serum were incubated at 37 °C and bacterial counts were determined by plate count method at 1, 2, 4, 8, 12 and 24 h. The limit of detection was 10 cfu/mL.

### PK and PD integration and modeling

By using in vitro MIC and in vivo PK parameters, the surrogate markers of antimicrobial activity (AUC/MIC) were determined for serum after IM dosing of tylosin for each pigs. Results were expressed as means ± SD.

The relationship between the ex vivo AUC_24_/MIC and log_10_ difference between the initial bacterial count (in number of per mL) and the bacterial count after 24 h of incubation was established for serum by using the Sigmoid *E*_max_ model, this model was described by the following equation:

$$ E={E}_0-\frac{\left({E}_{\mathrm{max}}-{E}_0\right)\cdotp {C_e}^N}{{EC_{50}}^N+{C_e}^N} $$

In which E is the antibacterial effect measured as the change in the bacterial count (log_10_ cfu/mL) in the serum sample after 24 h of incubation compared to the initial log_10_ cfu/mL, *E*_*max*_ is the maximum antibacterial effect determined as difference in log_10_ cfu/mL in sample incubated between 0 h and 24 h, E_0_ is the change in log_10_ difference in bacterial count in the control sample between 0 and 24 h of incubation; EC_50_ is the AUC/MIC value producing 50% of the maximum antibacterial effect; C_e_ is the AUC/MIC in the effect compartment (the ex vivo site, that is serum); and N is the Hill coefficient, which describes the steepness of the AUC/MIC-effect curve.

Three levels of antibacterial effect of tylosin were quantified from the sigmoid *E*_max_ equation by determining AUC/MIC required for bacteriostatic action (no change in bacterial counts after 24 h incubation, E = 0); bactericidal action (a 99.9% reduction in bacterial count, E = − 3), and bacterial elimination (the lowest AUC/MIC that produce a 99.99% reduction in the count, E = − 4) in each of the serum.

#### Dosage regimen and resistance risk assessment

The calculation of the potential optimal dosage could be performed using this equation:

$$ Dose=\frac{\left({AUC}_{24}/ MIC\right)\times MIC\times CL}{fu\times F} $$

In which MIC is minimum inhibitory concentration in this study; AUC/MIC is the target end point for optimal efficacy; CL is clearance; fu is the free fraction of tylosin. In this study, tylosin is a moderately bound by serum proteins (40%) [33]. F is bioavailability.

For risk assessment different concentration of tylosin (0,0.125, 0.25, 0.5, 1, 2, 4, 8 μg/mL) were added to each tubes containing bacterial suspension 10^10^ cfu/mL. the colony were checked after 0, 2, 4, 12, 24 h incubation, Any plate containing 100 cfus or less was not considered to be a drug-induced mutation.

## Abbreviations

AUC: Area under the curve
AUC/MIC: The area under the curve divided by the MIC
AUMC: The area under the first moment curve
CFU: Colony forming unit
CLSI: Clinical and Laboratory Standards Institute
C_max_: Peak concentration
C_max_/MIC: The maximum concentration divided by MIC
HPLC: High performance liquid chromatography
LOD: The limits of detection
LOQ: The limit of quantification
MBC: The minimal bactericidal concentration
MH broth: Mueller hinton broth
MIC: The minimum inhibitory concentration
MPC: The mutant prevention concentration
MRT: The mean residence time
MSW: Mutation selection window
PAE: The post-antibiotic effect
PK/PD: Pharmacokinetics/pharmacodynamic
*S. suis*: *Streptococcus suis*
T_>MIC_: The time that tylosin concentrations were above the minimum inhibitory concentration
T_max_: Time to reach C_max_
TSA: Tryptone soybean agar
